# Maternal Forced Swimming Reduces Cell Proliferation in the Postnatal Dentate Gyrus of Mouse Offspring

**DOI:** 10.3389/fnins.2016.00402

**Published:** 2016-08-29

**Authors:** Frederick Wasinski, Gabriel R. Estrela, Aline M. Arakaki, Michael Bader, Natalia Alenina, Friederike Klempin, Ronaldo C. Araújo

**Affiliations:** ^1^Max Delbrueck Center for Molecular Medicine, Molecular Biology of Hormones in the Cardiovascular SystemBerlin, Germany; ^2^Department of Biophysics, Federal University of São PauloSão Paulo, Brazil; ^3^Charité - University Medicine BerlinBerlin, Germany

**Keywords:** exercise, neurogenesis, BrdU, pregnancy, hippocampus

## Abstract

Physical exercise positively affects the metabolism and induces proliferation of precursor cells in the adult brain. Maternal exercise likewise provokes adaptations early in the offspring. Using a high-intensity swimming protocol that comprises forced swim training before and during pregnancy, we determined the effect of maternal swimming on the mouse offspring's neurogenesis. Our data demonstrate decreased proliferation in sublayers of the postnatal dentate gyrus in offspring of swimming mother at postnatal day (P) 8 accompanied with decreased survival of newly generated cells 4 weeks later. The reduction in cell numbers was predominantly seen in the hilus and molecular layer. At P35, the reduced amount of cells was also reflected by a decrease in the population of newly generated immature and mature neurons of the granule cell layer. Our data suggest that forced maternal swimming at high-intensity has a negative effect on the neurogenic niche development in postnatal offspring.

## Introduction

Continued neuron generation in the adult brain contributes to plasticity that allows immediate or long-term adaptations to neurogenic stimuli (Kempermann et al., 1997; van Praag et al., 1999). Physical exercise is an external factor that strongly induces the proliferation of precursor cells in the adult mouse hippocampus (van Praag et al., 1999; Kronenberg et al., 2003). The beneficial effect of physical activity lies in its proposed use as non-pharmacotherapy to increase mood and to ameliorate the outcome of neurological diseases (Ahlskog et al., 2011; Klempin et al., 2013; Gomes et al., 2014). In animal models, forced or voluntary exercise can be distinguished. Several studies reveal positive effects of voluntary running such as improved metabolism, memory, or increased adult neurogenesis in rodents (van Praag et al., 1999; Muller et al., 2011; Inoue et al., 2015). Furthermore, maternal running exercise reveals beneficial effects on metabolic health of offspring (Carter et al., 2013; Stanford et al., 2015). However, inadequate duration or intensity may lead to excessive stress associated with increased corticosterone levels (Girard and Garland, 2002; Contarteze et al., 2008) that may in turn decrease adult neurogenesis (Wong and Herbert, 2006).

More recently, studies focus on swimming exercise as it improves the immune system and has neuroprotective effects (Wasinski et al., 2013; Goes et al., 2014); the effect on hippocampal neurogenesis has not yet been shown. In humans, one advantage of moderate swimming as compared to running is continued training during pregnancy. In animal models, we and others have shown that maternal swimming exercise is beneficial for the mother as well as the offspring's metabolism, it increases glucose tolerance and induces mitochondrial biogenesis (Damasceno et al., 2013; Marcelino et al., 2013; Wasinski et al., 2015). However, the effect of maternal swimming on brain plasticity in the offspring has not yet been studied. In humans, continued physical activity during pregnancy showed advanced neurobehavioral maturation in children (reviewed Clapp, 2000). A pro-mitotic effect in the postnatal hippocampus of mouse offspring following maternal voluntary wheel running has been shown (Bick-Sander et al., 2006). Here, we studied the effect of maternal swimming exercise on early postnatal cell proliferation in the dentate gyrus (DG) of mouse offspring. We found that maternal swimming as opposed to voluntary wheel running decreased the number of mitotic cells in the DG at P8, that led to decreased numbers of newly generated cell survival at P35. The reduced amount of cells was predominantly seen in the immature and mature neuron population.

## Materials and methods

### Animals

**Figure 1:**
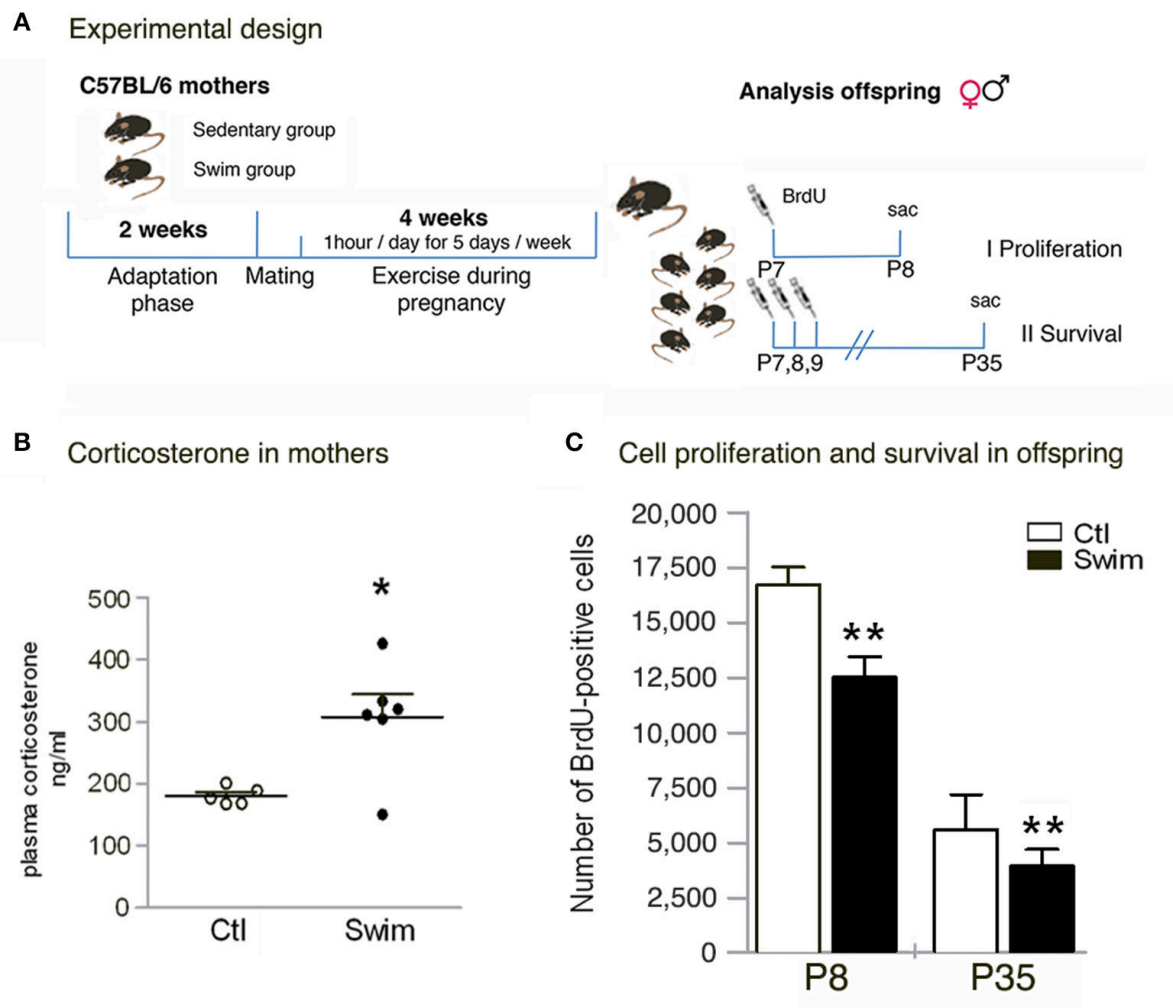
**Quantitative analysis of corticosterone levels in mothers, and absolute numbers of BrdU- positive cells in the pup's postnatal dentate gyrus. (A)** Experimental design. Female mice are either subjected (Swim group) or not subjected (Sedentary group, Ctl) to a training adaptation phase for 2 weeks followed by mating (approx. 1–5 days). The swimming group continues exercise during pregnancy (1 h per day, 5 days per week) for 18–21 days (depending on the length of pregnancy). Offspring of both groups is randomly split into two groups to determine I) proliferation and II) survival. **(B)** Corticosterone analysis in mothers at day 20 of pregnancy reveals significant increased levels following maternal swimming. **(C)** When determining proliferation (P8) and survival (P35), the number of BrdU+ cells is significantly reduced in the dentate gyrus of pups from swimming mothers. Student's *t* test *p < 0.05, **p < 0.01 between Ctl and Swim; data are presented as mean ± SEM.

Female C57BL/6 mice (8–12 weeks old) were obtained from the Animal Care Facility at the Federal University of São Paulo (UNIFESP). Animals (*n* = 14, 7 per group) were single housed under standard conditions with access to water and food *ad libitum*. All procedures were previously reviewed and approved by the internal ethical committee of the institution. One group of mice was subjected to physical swimming through a period of 6 weeks; the other group did not exercise (sedentary group / Ctl). Briefly, after a 2-week-adaptation period for the exercising group, female animals were mated with males of the same strain; the swimming assigned group continued training in the meantime, and during pregnancy (4 weeks including mating; Figure 1A).

To study postnatal neurogenesis in the offspring, male and female pups from either sedentary or trained mothers were split randomly into two groups to determine cell proliferation (at P8) and survival of newly generated cells (P35). Bromodeoxyuridine (BrdU, 50 mg/Kg body weight; Sigma) was injected intraperitoneal on P7 to assess cell numbers 24 h later (P8, *n* = 24), and ones per day on P7, P8, and P9 to examine cell survival and differentiation 4 weeks later (P35, *n* = 22).

### Swimming exercise

One group of female mice was subjected to swimming sessions in a swimming system adapted for mice (at 30°C) (Evangelista et al., 2003). Briefly, the 300-liter tank consists of 10 lanes fitted with air pumps to maintain mice in constant motion. Swimming was done during consecutive weekdays with 2 days off over the weekend. The adaptation period comprised 14 days with initial swimming sessions of 20 min per day during the first week (day 1–5). The time gradually increased until mice were able to swim for 60 min per day at “moderate intensity” induced by a load (3% of the body weight attached to the tail (Wasinski et al., 2013). Following 1–5 days of mating, pregnant mice were exercising 60 min per day (18–21 days, corresponding to the last 3 weeks of the total exercise period, Figure 1A); however, during the last week of pregnancy the load was removed. The presence of a vaginal plug was used to indicate pregnancy and considered as the first day of gestation (Figure 1A). Swimming was stopped immediately when the pups were born.

### Corticosterone levels

In the morning of day 20 of pregnancy, blood was collected from the heart shortly before perfusion of ketamine/xylazine-anesthesized mothers (another set of animals, *n* = 6 per group for swimming and sedentary control). Blood serum was obtained by centrifugation at 10,000 rpm for 15 min at −80 degrees; corticosterone levels were measure with a commercial enzyme-linked immunosorbent assay (ELISA, IBL international, Germany).

### Immunohistochemistry

For proliferation, mice were decapitated at P8 and the brains were transferred into 4% paraformaldehyde (PFA) over night. For survival of newly generated cells, P35 offspring was deeply anesthetized with ketamine/xylazine (10 ml/Kg body weight) and perfused transcardially with 0.9% sodium chloride followed by 4% PFA. Brains were removed from the skulls, postfixed in PFA at 4°C over night, and transferred into 30% sucrose (dissolved in 0.1 M phosphate buffer). Sequential 40 μm coronal sections were cut on a cryostat (Leica CM 3050, Germany) and cryoprotected. For BrdU staining, DNA was denatured in 2N HCl for 20 min at 37°C. Sections were then rinsed in 0.1 M borate buffer and washed in Tris-buffered saline (TBS). Sections were stained free-floating with all antibodies diluted in TBS containing 3% donkey serum and 0.1% Triton X-100.

Primary antibodies were applied in the following concentrations: anti-BrdU (rat, 1:500; Biozol), anti-calbindin D-28k (rabbit, 1:000; Swant), anti-doublecortin (DCX; goat, 1:250; Santa Cruz Biotechnology), anti-NG2 (rabbit, 1:1000; Santa Cruz Biotechnology), anti-S100β (mouse, 1:2000; Acris Antibodies). For immunofluorescence, Alexa488-conjugated, Cy3-conjugated, or Alexa647-conjugated secondary antibodies (Jackson ImmunoResearch Laboratories) were used at a concentration of 1:250. Fluorescent sections were coverslipped in Vector shield (Vector Laboratories).

### Quantification

One-in-six series of sections of each brain were stained, and BrdU-immunoreactive cells were counted throughout the rostro-caudal extent of the DG, and with respect to the different layers (40x, Keyence microscope). Results were multiplied by six to obtain the total number of BrdU-positive cells per DG. A one-in-six series of adjacent sections was further used to measure DG volume at P35. First, the area of the DG was traced using a semiautomatic area measurement system (10x, Keyence microscope). The volume was determined by summing the traced areas per slice multiplied by the distance between slices (240 μm). For phenotypic analysis, fifty to 100 randomly selected cells per animal were co-stained for markers of specific lineages and evaluated by examining orthogonal views from a series of focal planes using a Leica TCS SP5 (Leica) confocal microscope.

### Statistical analysis

Student's *t* test was used for individual pair-wise comparisons. To detect differences between group means, one-way analysis of variance (ANOVA) was performed (PRISM software). All values are expressed as mean ± SEM. *P* < 0.05 were considered statistically significant.

## Results

We applied daily swimming to pregnant mice to determine the effect on the offspring's postnatal neurogenesis (Figure 1A). Beforehand, corticosterone was measured in mothers (20th day of pregnancy) in the morning after the last swimming session to detect stress levels following continued exercise. Blood serum of exercising mice revealed a significant increase in corticosterone levels compared to sedentary pregnant controls (Ctl 180.6 ± 14.1 ng/ml vs. Swim 307.6 ± 88.9 ng/ml, *p* = 0.012; Figure 1B). When examining the offspring, no changes in pup size, sex ratio, or bodyweight of the pups at P8 and P35 were observed (data not shown). We next assessed the number of proliferating precursor cells at P8, 24 h after one injection of BrdU. Our data revealed significantly less BrdU-labeled cells in the entire DG of mice from swimming mothers (Ctl 16,730 ± 776 vs. Swim 12,540 ± 918, *p* = 0.0030; Figure 1C). At P35, the reduction in the number of BrdU-positive cells was maintained in mice of swimming mothers compared to control (*p* = 0.010; Figure 1C). Although, less proliferating cells were found after maternal swimming, no difference has been observed in the volume of the DG at P35 (Ctl 0.806 ± 0.071 vs. Swim 0.855 ± 0.063 mm^3^, *p* = 0.631).

**Figure 2:**
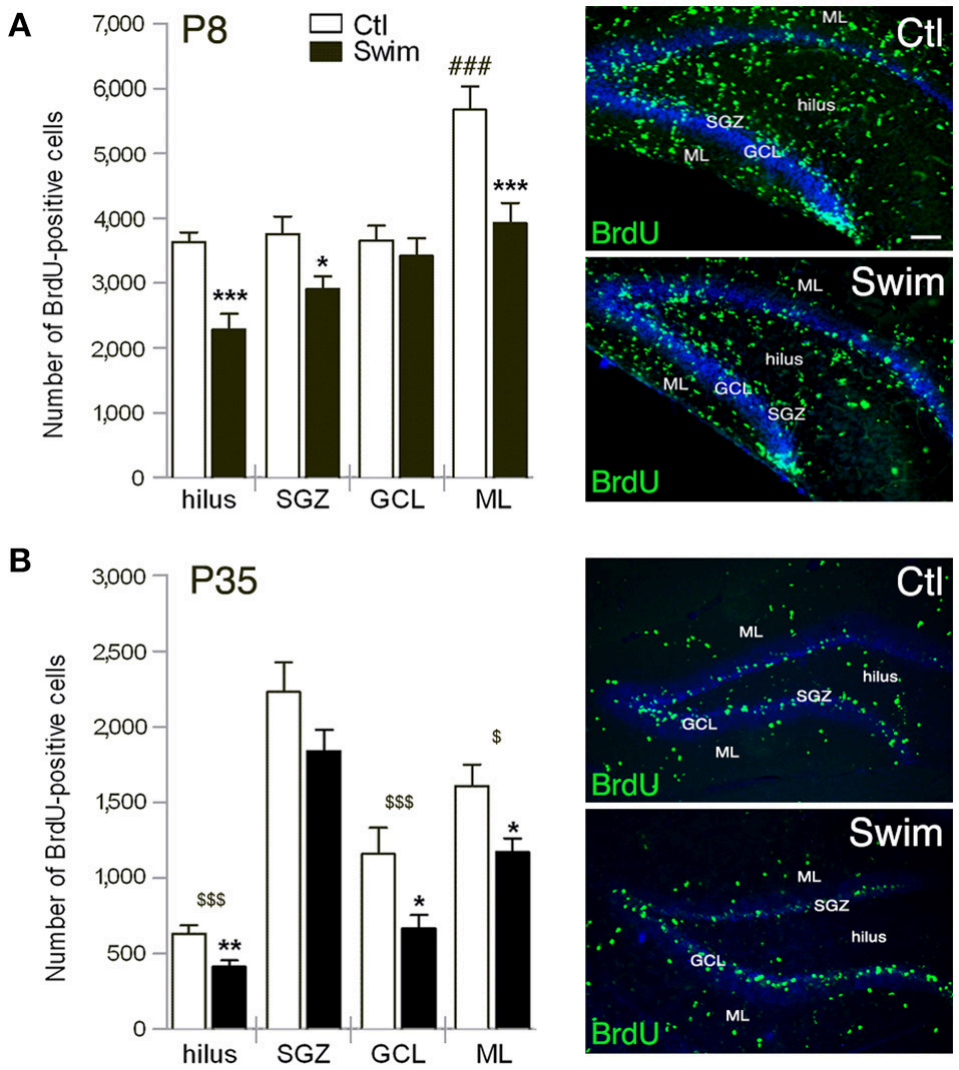
**Quantification of BrdU-positive cells in sublayers of the postnatal dentate gyrus (DG) at P8, 24 h after one injection of BrdU (A), and at P35, 4 weeks after three daily injections of BrdU (B)**. **(A,B)** The hilus and molecular layer (ML) of pups of swimming mothers (Swim) reveal significant less numbers compared to sedentary control (Ctl). At P8 the number is also reduced in the subgranular zone (SGZ), at P35 in the granule cell layer (GCL) of the swimming group. Scale bar = 100 μm. Student's *t* test *p < 0.05, **p < 0.01, ***p < 0.001 indicates statistical significance relative to Ctl; One-way ANOVA ^*###*^*p* < 0.001 to all layers of sedentary group Ctl; in **(A)**, and ^$^*p* < 0.05, ^$$$^*p* < 0.001 relative to SGZ for each group in **(B)**; data are presented as mean ± SEM.

We next determined which layer in the postnatal DG of P8 mice might reflect the decrease and found significantly reduced numbers (approximately one-third) of proliferating precursor cells in the hilus (*p* = 0.0002), and molecular layer (ML, *p* = 0.0014); fewer cells were also found in the subgranular zone (SGZ, *p* = 0.0156) with unchanged numbers in the granule cell layer (GCL; Figure 2A). Notably, the majority of BrdU-positive cells in the sedentary group was found in ML (One-way ANOVA to SGZ, GCL, and hilus, respectively, *p* < 0.001) with similar cell numbers observed for all other layers in the control groups. Spatial distribution of surviving precursor cells at P35, 4 weeks after three daily injections of BrdU, revealed significantly fewer cells in the hilus (*p* = 0.006) and ML (*p* = 0.022) of pups born of swimming mothers as was observed at P8 (Figure 2B). Noticeable, at P35 the number of BrdU-positive cells in the GCL was highly reduced in mice of exercising mothers in comparison to sedentary controls (*p* = 0.027; Figure 2B). Majority of proliferating cells for both experimental groups was found in the SGZ with respect to the neurogenic niche in the adult. Cell numbers of the SGZ were reduced in comparison to P8 by only a third while survival of proliferating cells was highly decreased in all other sublayers from P8 to P35.

Next, we assessed the phenotype of newly generated cells in the SGZ and GCL at P35 by co-labeling with markers of the neuronal and glial lineages. Our data revealed that only a very small population of cells coexpress BrdU and the oligodendrocyte precursor marker Neuron-Glia (NG)2 or the astroglia marker S100β as expected, with no difference seen between the experimental groups (Table 1). However, the decrease in the number of BrdU-positive cells was reflected by a significant reduction in the fraction of immature (BrdU/DCX-positive) and mature (BrdU/calbindin-positive) neurons in the SGZ and GCL of offspring of swimming mothers (*p* = 0.031 and *p* = 0.023, respectively; Table 1).

**Table 1:** **Number and phenotypes of BrdU-positive cells in the SGZ/GCL at P35**.

	**Cell number**	**DCX (%)**	**Calbindin (%)**	**S100ß (%)**	**NG2 (%)**	**Other (%)**
Control	3382 (535)	23.1 (3.4)	35.6 (1.2)	2.7 (0.8)	5.2 (1.0)	33.4
Swim	2499 (586)	12.9 (1.3)^*^	27.8 (2.4)^*^	1.9 (0.2)	5.0 (1.7)	52.4

*The percentages of BrdU-positive cells co-labeled for DCX (immature neurons), Calbindin (mature granule cells), S100β (astrocytes), NG2 (oligodendrocyte precursors) or neither marker are presented. All data are presented as mean (standard error)*.

* *p < 0.05 statistical significance between Control and Swim*.

## Discussion

Our data demonstrate that maternal “high-intensity” swimming exercise decreases proliferation and survival of newly generated cells in the DG of postnatal offspring. Reduced numbers predominantly found in the hilus and ML reflected the overall decrease. At P35, the reduction was mainly seen in the number of newly generated cells of the GCL expressing DCX and calbindin. Our data suggest that forced swimming exercise during pregnancy has a negative effect on the neurogenic niche development in postnatal offspring.

Voluntary wheel running has been established to augment neurogenesis in rodents (van Praag et al., 1999; Kronenberg et al., 2003). Soluble factors accompany this effect such as growth- and neurotrophic factors, or neuromodulators (Trejo et al., 2001; Fabel et al., 2003; Moon et al., 2012; Klempin et al., 2013). An earlier study also showed stimulation of postnatal DG development in the offspring of maternal voluntary wheel running (Bick-Sander et al., 2006). Maternal swimming in contrast, appears to be a bad exercise for the pup's brain development. A positive outcome on brain maturity as well as metabolism depends on frequency, duration, and intensity of the exercise (Ploughman et al., 2007; Speck et al., 2014). Swimming is rather forced work out for rodents and studied as aerobic exercise to challenge the metabolism and immune system (Wasinski et al., 2013; Cunha et al., 2015). Only few studies address the effect of swimming exercise on the brain revealing a neuroprotective role in Parkinson's disease, and increased neurotrophin levels (Goes et al., 2014; Jiang et al., 2014). There is no literature on “voluntary” swimming; however, different swimming protocols have been used comprising of either low- or high-intensity exercise distinguished by the length and intensity of the swimming (10, 30, 60, or 120 min per day for 2 to 8 weeks) (Lee et al., 2006; Radak et al., 2006; Marcelino et al., 2013; Wasinski et al., 2015) that revealed beneficial effects on both, brain and periphery. We have recently shown that swimming prior and during pregnancy up to 1 h per day/5 days per week positively affect glucose tolerance (Wasinski et al., 2015). Nevertheless, it seems no benefits occur for the postnatal hippocampus in the offspring using the same high-intensity protocol, as the population of immature and mature neurons is decreased considering more DCX-, and calbindin-positive cells are better.

Exhaustive exercise may lead to stress and increased corticosterone levels (Contarteze et al., 2008). Our protocol forces exercise at consistent times and intensity and we found elevated serum corticosterone levels in highly pregnant female mice following 6 weeks of swimming. Pregnancy itself is associated with the attenuation of the HPA-Axis (reviewed in Slattery and Neumann, 2008) affecting the offspring development (reviewed in Douglas, 2010). Prenatal stress can also reduce postnatal neurogenesis (Lucassen et al., 2009); yet, there are mechanisms that protect the offspring against stress-induced maternal corticosterone levels (Thomas et al., 2007). We did not determine glucocorticoid levels in the pups as they may also affect postnatal cell genesis. Other studies in contrary show that early life-stress like maternal deprivation did not affect adult neurogenesis (Herpfer et al., 2012). Furthermore, when (maternal) exercise was done at lower duration and speed, a similar increase in corticosterone levels was observed which did not negatively affect neurotrophic factor levels and even increased the offspring's performance in the Morris water maze in rats (Ploughman et al., 2007; Akhavan et al., 2008). These data reveal that forced exercise at lower intensity does not harm brain plasticity. A new study claims that treadmill running has a positive effect on the offspring brain development by increased BDNF levels (da Silva et al., 2016).

The hilus is the source of granule cells in late development of the neurogenic niche (Altman and Bayer, 1990). In pups of swimming mothers, BrdU cells in the hilus were reduced at both time points together with decreased cell numbers in the SGZ (at P8). This might explain the reduced number of cells in the GCL 4 weeks later (at P35) where to newly generated cells migrate out of the SGZ. However, the majority of newly generated cells at P35 for both experimental groups (w/o statistical difference) was found aligned in the SGZ shaping the neurogenic niche; thus this continues source of granule cells is still intact. In addition to being less, the distribution of immature and mature newborn neurons among newly generated cells was decreased. We tested other cell fate markers and observed no changes for NG2 or S100β expression; Sox2 was not visible. Considering the protocol in our study being stressful, it largely affected the number of proliferating cells in the hilus and ML with considerable effects on the postnatal neurogenic niche. Further experiments will define the impact on brain function, and cognitive processes later in life since changes in hippocampal neurogenesis due to stress maybe recovered in adulthood (Herpfer et al., 2012).

These data are the first to determine the effect of maternal swimming on the offspring's postnatal hippocampal neurogenesis. Using the same high-intensity swimming exercise protocol, it beneficially influences the metabolism of adult offspring (Wasinski et al., 2015) whereas it negatively affects newly generated cells in the postnatal neurogenic niche DG.

## Author contributions

FW, FK, and RA designed research; FW, GE, AA, and FK performed research and analyzed the data; FW and FK wrote the paper. FW, GE, AA, MB, NA, FK, RA reviewed the manuscript.

### Conflict of interest statement

The authors declare that the research was conducted in the absence of any commercial or financial relationships that could be construed as a potential conflict of interest.
